# Supplementary material for: Long-read sequencing identifies FGF14 repeat expansions in Parkinson’s disease
Source: medRxiv. 2025 Aug 19:2025.08.14.25333596. Preprint. [Version 1] doi: 10.1101/2025.08.14.25333596 (PMC12393589; doi:10.1101/2025.08.14.25333596)
Supplement: 1 [file NIHPP2025.08.14.25333596V1-supplement-1.pdf]

medRxiv preprint doi: https://doi.org/10.1101/2025.08.14.25333596; this version posted August 19, 2025. The copyright holder for this preprint (which was not certified by peer review) is the author/funder, who has granted medRxiv a license to display the preprint in perpetuity.
It is made available under a CC-BY 4.0 International license .

Supplementary Figures

Supplementary Figure 1. Integrative Genomics Viewer visualization of the *FGF14* (GAA)□ repeat expansion in affected carriers.
Genome browser snapshots showing aligned long-read sequencing reads at the *FGF14* locus for the five PD patients carrying the pathogenic expansion. Expanded alleles are indicated by increased repeat length relative to the reference.

Supplementary Figure 2. Pathogenic and reduced penetrance *FGF14* (GAA)□ repeat expansions identified in PPMI Parkinson's disease patients. a) Waterfall plot displaying the repeat lengths observed in five Parkinson's disease cases carrying fully penetrant (GAA)□ expansion ≥300 repeat units, **b)** three patients and three healthy controls with reduced penetrance *FGF14* (GAA)□ ≥ 250 repeat units, **c)** one patient with (GAAGCA)□ motif, **d)** two controls with (GAAGGA)□ expansions.

Supplementary Figure 3. Distribution of age at onset by *FGF14* (GAA)□ repeat length. Analysis of five *FGF14*-GAA expansion carriers from the PPMI cohort (left), and analysis of all PPMI Parkinson's disease cases with available age at onset and repeat length data (right).

Supplementary Figure 4. Haplotype-specific DNA methylation across the *FGF14* (GAA)□ expansion a–e) Methylation frequency plots generated with modbamtools for PD patients heterozygous for a pathogenic *FGF14* (GAA)□ expansion, based on PPMI blood-derived long-read sequencing data. **f)** Methylation frequency plot for the control individual from the NABEC cohort, based on adaptive sampling from cerebellum tissue. Haplotypes are phased, with haplotype 1 corresponding to the non-expanded allele and haplotype 2 representing the expanded allele. Methylation frequency is shown above, with the *FGF14* gene structure overlaid. Individual reads are shown below, with blue indicating hypomethylation and red indicating hypermethylation.

medRxiv preprint doi: https://doi.org/10.1101/2025.08.14.25333596; this version posted August 19, 2025. The copyright holder for this preprint (which was not certified by peer review) is the author/funder, who has granted medRxiv a license to display the preprint in perpetuity.
It is made available under a CC-BY 4.0 International license .

Supplementary Table 1. Demographic characteristics of study participants (PPMI, All of Us, HBCC, NABEC, 1000 Genomes Project).

Supplementary Table 1. Demographic characteristics of study participants (PPMI, All of Us, HBCC, NABEC, 1000 Genomes Population).

		N	F:M ratio	Mean age	Mean age at diagnosis	Ancestry %
PPMI	cases	411	0.45	61.5 ± 10.0	61.0 ± 9.6	94% EUR, 1.7% EAS, 1% AMR, 0.7% CAH, 0.7% AAC, 0.2% AFR, 0.2% SAS, 0.2 %MDE
	controls	197	0.71	61.1 ± 10.4		94% EUR, 2% AAC, 1.5% AFR, 1.5% AMR, 0.5% SAS
All of Us	controls	184	1.14	55.4 ± 18.7		100% EUR
HBCC	controls	133	0.64	45.1 ± 14.3		47% AAC, 53% AFR
NABEC	controls	204	0.56	52.7 ± 27.5		99% EUR, 1% AJ
1000 Genomes Project	controls	908	1.00	NA		27% AFR, 19% EAS, 19% SAS, 18% EUR, 17% AMR

AAC= African admixed ancestry; AFR= African ancestry; AJ: Ashkenazi Jewish ancestry; AMR: American ancestry; CAH: Complex Admixture History; EAS: East Asian ancestry; EUR: European ancestry; MDE: Middle Eastern ancestry; SAS: South Asian ancestry; F: female; M :male; N: number; NA: not available

Supplementary Table 2. Summary of *FGF14* repeat expansion in 411 PPMI cases and 1,626 controls from PPMI, NABEC, HBCC, All of Us, and 1,000 Genomes Project participants. Length is defined by the average of the ten longest alleles carrying the expansions.

medRxiv preprint doi: https://doi.org/10.1101/2025.08.14.25333596; this version posted August 19, 2025. The copyright holder for this preprint (which was not certified by peer review) is the author/funder, who has granted medRxiv a license to display the preprint in perpetuity.
It is made available under a CC-BY 4.0 International license .

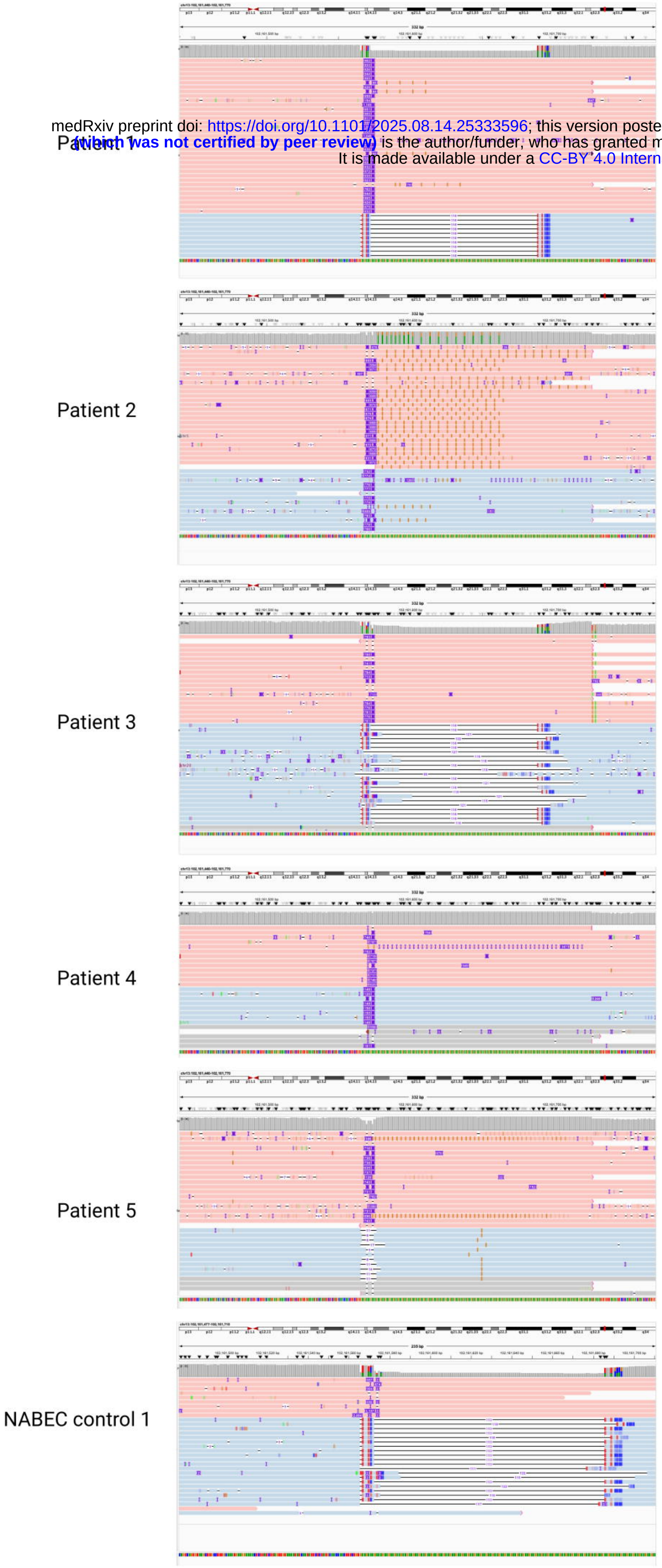

medRxiv preprint doi: https://doi.org/10.1101/2025.08.14.25333596; this version posted August 19, 2025. The copyright holder for this preprint (which was not certified by peer review) is the author/funder, who has granted medRxiv a license to display the preprint in perpetuity.
It is made available under a CC-BY 4.0 International license .

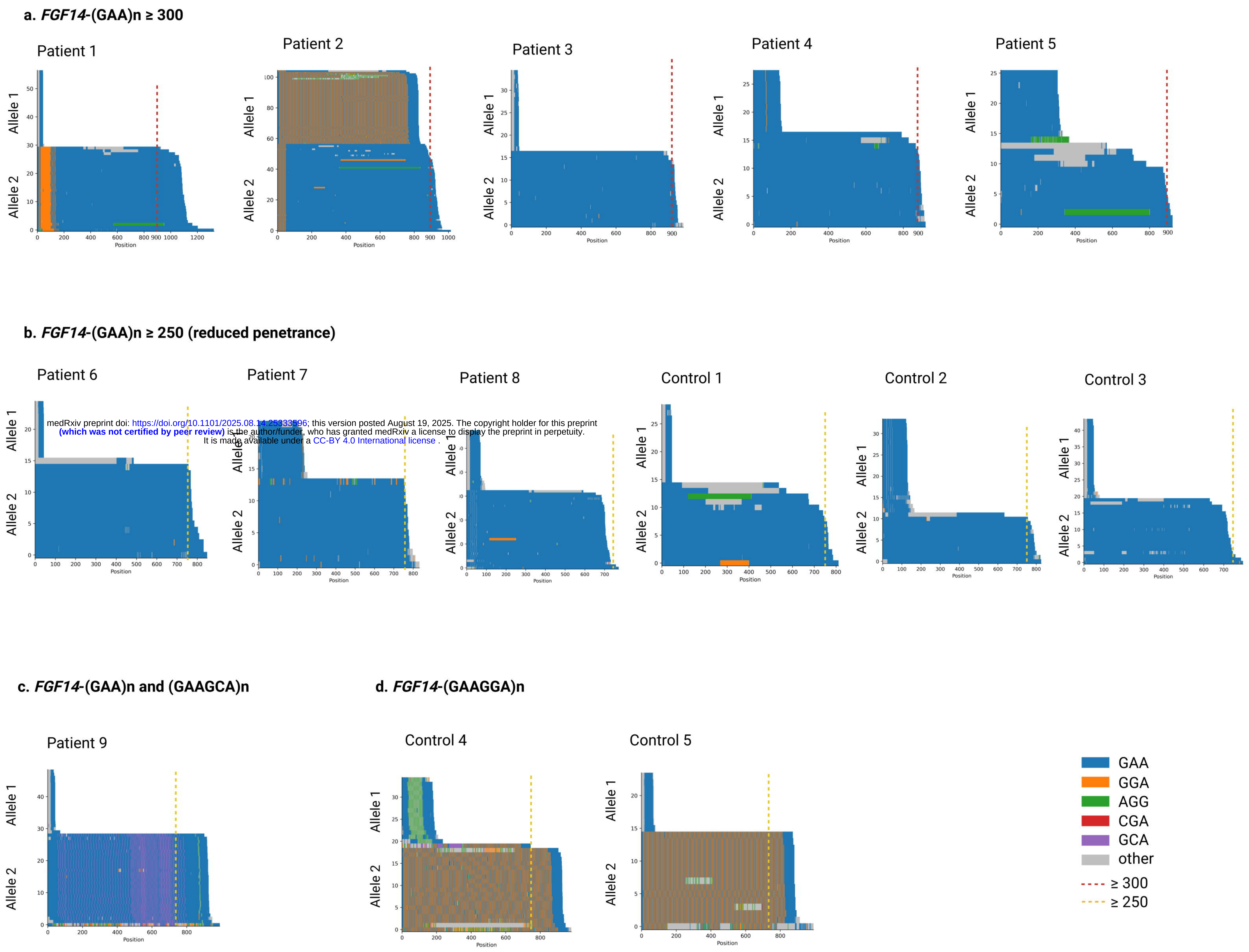

Supplementary Figure 3

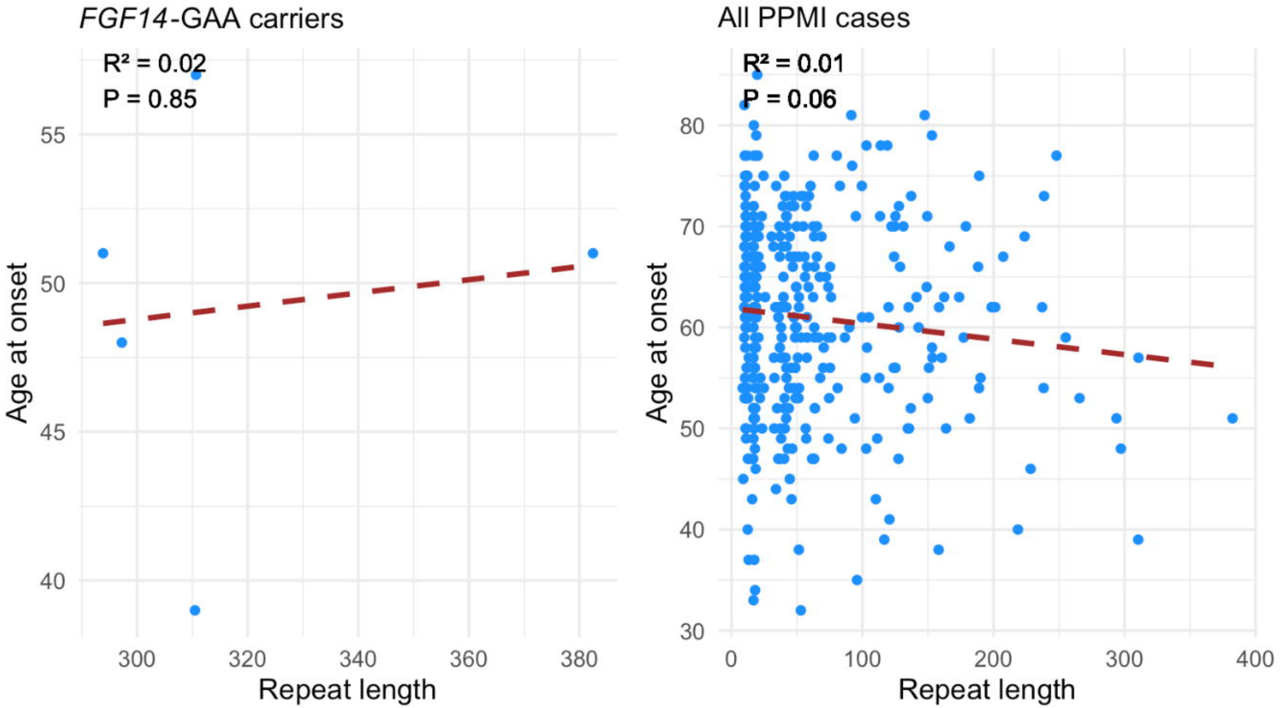

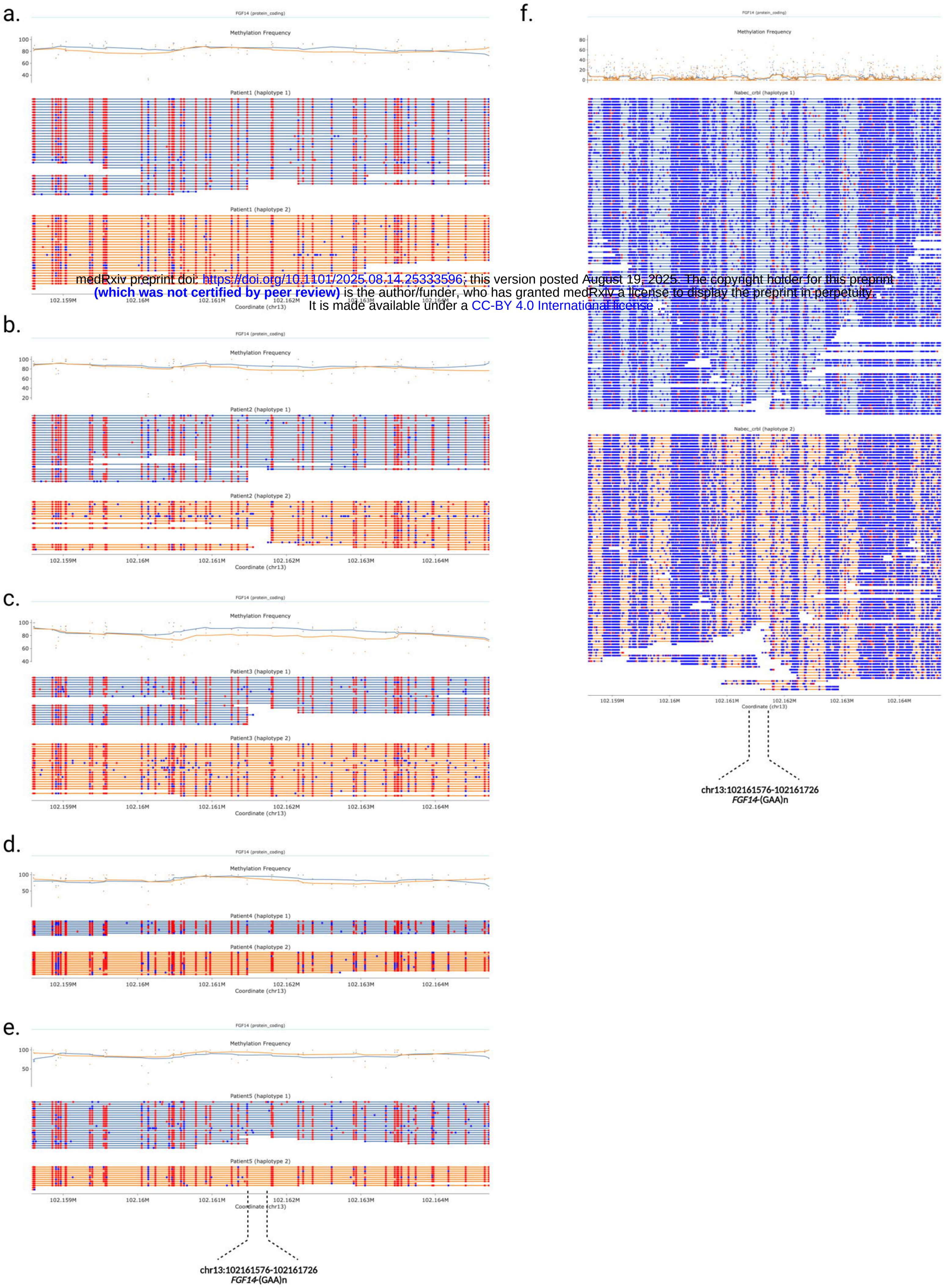

medRxiv preprint doi: https://doi.org/10.1101/2025.08.14.25333596; this version posted August 19, 2025. The copyright holder for this preprint (which was not certified by peer review) is the author/funder, who has granted medRxiv a license to display the preprint in perpetuity.
It is made available under a CC-BY 4.0 International license .
